# Application of Decision-Tree-Based Machine Learning Algorithms for Prediction of Antimicrobial Resistance

**DOI:** 10.3390/antibiotics11111593

**Published:** 2022-11-10

**Authors:** Muhammad Yasir, Asad Mustafa Karim, Sumera Kausar Malik, Amal A. Bajaffer, Esam I. Azhar

**Affiliations:** 1 Special Infectious Agents Unit, King Fahd Medical Research Center, King Abdulaziz University, Jeddah 21589, Saudi Arabia; 2 Department of Medical Laboratory Sciences, Faculty of Applied Medical Sciences, King Abdulaziz University, Jeddah 21589, Saudi Arabia; 3 Graduate School of Biotechnology, College of Life Sciences, Kyung Hee University, Yongin 17104, Republic of Korea; 4 Department of Bioscience and Biotechnology, The University of Suwon, Hwaseong 18323, Republic of Korea

**Keywords:** machine learning, antimicrobial resistance, *Pseudomonas aeruginosa*, transcriptomics

## Abstract

Timely and efficacious antibiotic treatment depends on precise and quick in silico antimicrobial-resistance predictions. Limited treatment choices due to antimicrobial resistance (AMR) highlight the necessity to optimize the available diagnostics. AMR can be explicitly anticipated on the basis of genome sequence. In this study, we used transcriptomes of 410 multidrug-resistant isolates of *Pseudomonas aeruginosa*. We trained 10 machine learning (ML) classifiers on the basis of data on gene expression (GEXP) information and generated predictive models for meropenem, ciprofloxacin, and ceftazidime drugs. Among all the used ML models, four models showed high F1-score, accuracy, precision, and specificity compared with the other models. However, RandomForestClassifier showed a moderate F1-score (0.6), precision (0.61), and specificity (0.625) for ciprofloxacin. In the case of ceftazidime, RidgeClassifier performed well and showed F1-score (0.652), precision (0.654), and specificity (0.652) values. For meropenem, KNeighborsClassifier exhibited moderate F1-score (0.629), precision (0.629), and specificity (0.629). Among these three antibiotics, GEXP data on meropenem and ceftazidime improved diagnostic performance. The findings will pave the way for the establishment of a resistance profiling tool that can predict AMR on the basis of transcriptomic markers.

## 1. Introduction

Increased resistance in pathogens toward antimicrobials is a major threat to public health and development. Increasing resistance hinders the usage of traditional antimicrobials and leads to higher rates of ineffective and unsuccessful empiric antibiotic therapy [1]. If not effectively cured, diseases give rise to suffering, indisposition, and finally loss of life, and impose a huge economic burden on society and healthcare systems. The rapid spread and rise in AMR is usually driven by different factors which include misuse, overreliance, and overuse of antibiotics in agriculture and clinical settings [2]. Regardless of growing medical need, the antibiotic pipeline has slowed to a trickle over the last 20 years. Disturbingly, there are merely a few antimicrobial agents in the development phase to treat infections caused by multidrug-resistant pathogens [1].

*Pseudomonas aeruginosa* (*P. aeruginosa*) is Gram-negative, encapsulated, aerobic, rod-shaped pathogen, and is an opportunistic organism. It causes different acute and chronic persistent infections such as urinary tract infections, dermatitis, respiratory system infections, soft-tissue infections, and bacteremia [3]. It also causes a range of systemic infections, mostly in cancer patients, victims of severe burns, and in AIDS patients who are immunosuppressed [3].

To treat different bacterial infections empirically, physicians prescribe standard antimicrobials for treatment. Hence, it is very critical and important to investigate the resistance profile of antimicrobials before the treatment of bacterial infections. In terms of clinical perspective, rapid diagnostics are very important for the improvement of patient care because diagnostic tests are an important component in healthcare practice. Additionally, for antimicrobial susceptibility testing, we still rely on culture-based techniques, however, these conventional microbiology diagnostics are slow and labor intensive. In conventional microbiology methods, microbial growth, isolation, taxonomic identification, and antibiotic MIC tests take a lot of effort and time (i.e., more than 24 h for MRSA and months for tuberculosis) [4]. With these problems, physicians are left uncertain about the best drugs to prescribe to infections [5]. Moreover, this time interval also contributes to the rise and further spread of antimicrobial resistance.

*Pseudomonas aeruginosa* is clinically significant bacteria with inherent resistance to several antimicrobials [3]. This inherent multidrug resistance arises from the synergy be-tween less outer membrane permeability and particular antibiotic efflux pumps [6]. In carbapenems, resistance is usually facilitated by OprD loss, and because of this damage, *P*. *aeruginosa* exhibits resistance to imipenems. However, it confers a lesser degree of resistance in the case of meropenems [7]. In addition, the efflux systems that are involved to mediate the resistance to chloramphenicol, quinolone, and many other antimicrobials are also involved in carbapenem resistance. *Pseudomonas aeruginosa*, which express the MexEFOprN system or overexpress the MexAB-OprM system, show carbapenem resistance via repressing the transcription of oprD or pumping the drug out, respectively [8]. In addition to the OprD loss or drug efflux pumps, chromosomal AmpC β-lactamase plays an important role in carbapenem resistance in *P. aeruginosa* [9]. The usage of molecular diagnostics can serve as a substitute for culture-dependent techniques and can be very important in generating success in the fight against AMR. Identification and detection of genetic elements of AMR assure a great understanding of resistance mechanisms and can help in the timely reporting of susceptibility and resistance profiles of pathogens compared with conventional culture-dependent techniques. It has been reported that AMR can be very precisely predicted based on evidence resulting from the genome sequence [10]. However, in *P. aeruginosa*, even full genomic sequence data are scarce to predict AMR in isolates of clinical relevance [11]. This pathogen shows an intense phenotypic plasticity arbitrated by environment-driven malleable modifications in the transcriptional information. For instance, it acclimatizes to the presence of antimicrobials with the overexpression of the mex genes, encoding the antibiotic extrusion machineries. Likewise, high expression of the ampC-encoded intrinsic beta-lactamase confers AMR [11,12]. Hence, *P. aeruginosa* develops an environment-independent and self-sustaining resistance phenotype. In the present investigation, we explored whether AMR in *P. aeruginosa* can be reliably predicted using not just genomic imprinting data but also by employing quantitative GEXP data. Hence, drug-resistant clinical *P. aeruginosa* were used. 

We employed ten predictive ML models for the prediction of resistance to three commonly administered antibiotics for *P. aeruginosa* by training classifiers using quantitative GEXP information. We found that the use of information on the GEXP profiles can predict susceptibility and resistance in this particular bacteria with predictive values.

## 2. Results

We used data of 410 *P. aeruginosa* isolates and 10 ML models to predict the antibiotic resistance. The data retrieved from NCBI consisted of the sequences of genomic DNA, and by employing transcriptional profiles information on the GEXP, we used these data as an input to ML models. The isolates were susceptible and/or resistant to ciprofloxacin, ceftazidime, and meropenem (Figure S1). For each antibiotic, we included all respective isolates categorized as either “resistant” or “susceptible”. For each antibiotic, the training set comprised of 75% of the resistant and susceptible isolates, and 25% for the test set. In total, 10 machine learning classification methods were used on expression features for predicting resistance or susceptibility and the classifier performances were assessed. This study is a test case for AI4Water and was used as a classification problem. AI4Water is a useful and good tool and it employs a python-based framework for performing different tasks. 

**Figure 1:**
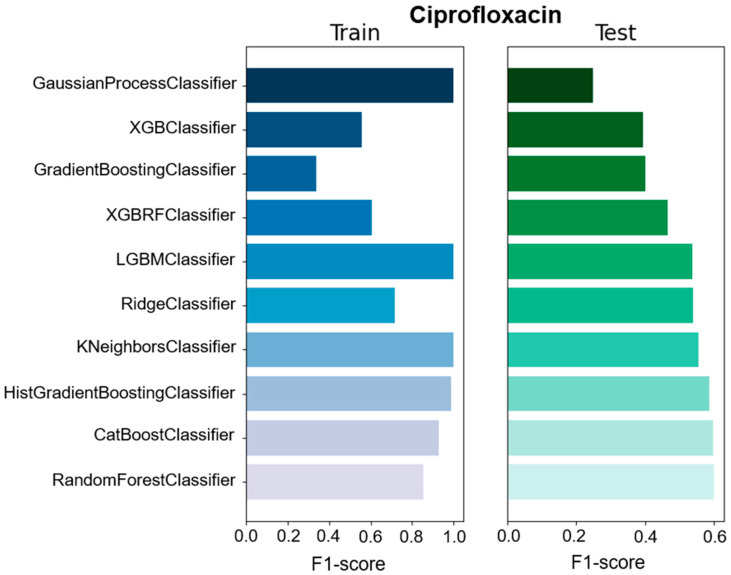
The comparison of F1-score of ten classification models used for gene expression prediction for ciprofloxacin. The blue color represents the training dataset and the green color represents the test dataset.

**Figure 2:**
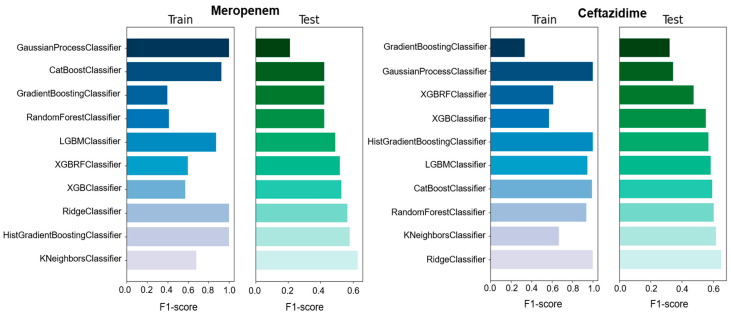
The comparison of F1-score of ten classification models used for gene expression prediction for meropenem and ceftazidime. The blue color represents the training dataset and the green color represents the test dataset.

We used different classifiers to calculate the predictive value of GEXP as well as the macro F1-score, accuracy, precision, and specificity as a general performance measure on the basis of classifiers trained on specific data types (Figure 1, Figure 2 and Figures S2–S8). The F1-score is one of the most important evaluation metrics in machine learning. In total we used ten classifier methods; CatBoostClassifier, GaussianProcessClassifier, GradientBoostingClassifier, HistGradientBoostingClassifier, KNeighborsClassifier, LGBMClassifier, RandomForestClassifier, RidgeClassifier, XGBClassifier, and XGBRFClassifier (Figure 1 and Figure 2 and Table S1). Among all the used ML models, four models showed high F1-score, accuracy, precision, and specificity compared with the other models. However, RandomForestClassifier showed moderate F1-score (0.6), precision (0.61), and specificity (0.625) for ciprofloxacin compared with other studies. In the case of ceftazidime, RidgeClassifier performed well and showed moderate F1-score (0.652), precision (0.654), and specificity (0.652) values. For meropenem, KNeighborsClassifier exhibited high F1-score (0.629), precision (0.629), and specificity (0.629) values (Table 1 and Table S1). Moreover, the sensitivity of 0.458 and predictive value of 0.8 to predict meropenem resistance and susceptibility based exclusively on GEXP data were high. For ceftazidime, the sensitivity (0.659) was higher than meropenem (0.458) on the basis of GEXP. Gene expression information considerably improved the performance of susceptibility and resistance sensitivity. 

**Figure 3:**
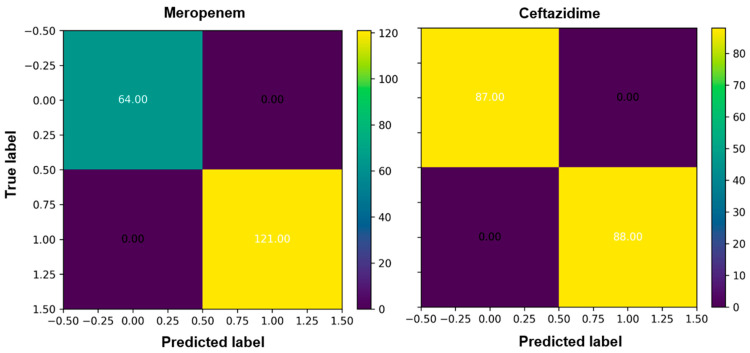
Confusion matrix for the gene expression values of *P. aeruginosa* isolates with a threshold set at 0.5. Rows represent the true transcriptomic values and columns represent the predictions.

We evaluated the prediction performance by confusion matrixes (CM). CM is used to narrate the performance and accuracy of a classification model on test data for which the true values are known. There are total of four standards in the CM (true negatives, false negatives, true positives, and false positives,) and can be used to compute numerous other metrics. The true negative (76.00) and true positive (84.00) scores for ciprofloxacin were high in the case of the RandomForestClassifier and the prediction was high (Figure 3, Figures S2 and S4). For ceftazidime, true the negative (87.00) and true positive (88.00) were higher with overall high prediction accuracy (Figure 3). 

The receiver operating characteristic (ROC) curve is another way to evaluate the performance of the model. In the ROC curve, the trade-off between the false-positive rate (equivalent to 1 minus specificity) and the true-positive rate (recall or sensitivity) is graphically displayed. We also used binary classifiers to evaluate with performance measures such as specificity, sensitivity, and performance as shown in the ROC plots. The true positive rate in the case of meropenem can be considered as a good classifier for a performance evaluation as shown in Figure 3. The nearer the curve is to the upper left place, the better the model is overall. The area under the curve summarizes how good a test is regardless of the threshold but does not define an operating model. Therefore, we must select the threshold to actually put the tool into practice.

**Figure 4:**
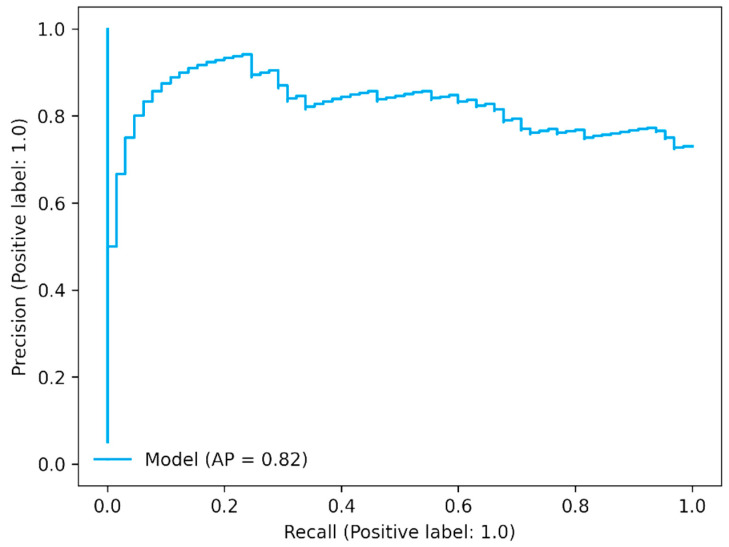
Precision–recall curve for meropenem.

The precision–recall curve shows the trade-off between precision (positive predictive value) and recall (sensitivity). It is also called a precision–recall (PR) curve. In the PR curve, the threshold defines a specific point on the PR curve, but it does not change the curve itself. The area under the curve is dependent on prediction because precision is dependent on prediction. In the case of three antibiotics, it showed good AP values as shown in Figure 4 for meropenem (Figure 4 and Figures S9–S11).

## 3. Discussion

Rapid diagnostics is very important in the fight against drug-resistant infections. As earlier, timely, and further comprehensive knowledge on antimicrobial resistance profile of pathogens has the ability to alter antibiotic prescribing conduct and improve patient outcomes. Because the treatment of various bacterial infections is carried out empirically, physicians recommend a standard antibiotic to patients. Hence, there is a growing awareness to obtain the results faster and this has introduced an investigation of molecular diagnostics substitutes to already available conventional microbiology methods (culture-based). Rapid diagnostics are very important to enhance patient care. Nevertheless, for the effective application of fast and dependable molecular tools, it is very important to recognize the entirety of the molecular determinants of resistance. In case of failure to identify resistance, it might elicit administration of suboptimal or ineffective antibiotic treatment and this constitutes a substantial risk, particularly in terminally ill people, and has dire consequences for patients. Overuse can also be a result to resistance and the unnecessary use of broad-spectrum antibiotics can be the consequence of resistance [1]. This puts patients at higher risks with severe side effects and drives hospital costs., and it can also substantially contribute to the development of drug resistance by applying undesired selective pressures [13,14]. We report that ML methods using transcriptomic and genomic features can offer extraordinary antimicrobial resistance assignment proficiencies for this particular bacteria. We observed that the performance of prediction was greatly dependent on the antibiotic.

We observed in the case of ciprofloxacin that sensitivity to predict resistance and susceptibility from GEXP data was high. Gene expression data can play an important role in the construction of a diagnostic system to test the resistance profile of bacteria. The idea can be of value if the information on GEXP is added as a fail-safe strategy [15]. To acquire maximum mean prediction accuracy, regression models were also used on the same samples; nevertheless, the mean accuracy and performance of classification models was more reliable than regression models [1,16,17]. 

Although this was a test case for AI4Water, our results on GEXP for three antibiotics were similar to the results reported by Khaledi et al. [18]. However, in the case of meropenem and ceftazidime, their sensitivity was high compared with these results. Hence, in the future, we and other researchers can work on improvements. 

Among the 10 used ML models, some of them had lower accuracy predictions. The machine learning modeling requires adequate input data to train the ML models to form a training dataset and a “testing dataset” to assess the performance of the model [1]. Among the three antibiotics, the resistant background of bacteria was different for each drug; therefore, after randomly splitting the limited data into a training dataset or testing dataset, different ML models did not have enough to learn from the training dataset which led to a relative lower accuracy while predicting the testing set of the model.

In conclusion, we demonstrate that using the transcriptomic features such as GEXP values is important for prediction and improving performance. Hence, susceptibility and resistance sensitivity are strongly dependent on the antibiotics. Moreover, analysis of the GEXP marker list revealed that the resistance phenotype in *P. aeruginosa* is complicated and not simple and that modifications in GEXP can change the resistance phenotype very significantly. 

The limitation of this study includes small sample size for bioinformatics and ML modeling analysis. In spite of high accuracy and good prediction of ML models, some improvement can be expected if larger sample sizes were used in this study.

## 4. Materials and Methods

### 4.1. Data Processing

In total, 410 clinical isolates of *P. aeruginosa* were used in this study. The data were collected from the National Center for Biotechnology Information (NCBI). All clinical isolates were tested for antibiotics susceptibility toward common antipseudomonas meropenem, ciprofloxacin, and ceftazidime. To analyze the sequences of isolates, we used SPAdes, v.3.0.1, to assemble the trimmed reads by using a particular parameter called as the careful parameter. We annotated genomes using Prokka, particularly the metagenome mode for gene calling. This method was used because genes present on resistance cassettes were frequently missed by the genome gene calling method. Moreover, Roary was used to cluster the gene sequences into gene families. 

### 4.2. Problem Description

In this study, we used observed gene expression data as the target value. The ML models were trained to predict the resistance profiles. Therefore, we solved a classification problem where the target of the ML model was to predict the gene expression values for three antibiotics.

### 4.3. Comparison between Machine Learning Models

In this study, ten ML models were used to check and compare the prediction accuracies of different models. We set the random seed parameter to 313 in order to maintain re-liability in the splitting of the dataset for comparative analysis of the results. Numerous ML models exist which can be used for solving a classification task. We tested the performance of ten models to predict the exact GEXP values for three antibiotics.

### 4.4. Train Test Split

We split the data into a 75% training set and a 25% test set. The splitting was conducted randomly to avoid any bias. The models were trained on the training set, while its accuracy was measured on the validation set. The aim of this process is to assess the ability of the ML algorithm to generalize to new data and select hyperparameters. After selecting the best hyperparameters for the model, we evaluated its performance on the test set. The test data were not seen by the model during training. In this way, the generalization ability of the model on the unseen data was assessed. 

### 4.5. Hyperparameter Optimization

The performance of the ML algorithm is greatly affected by the choice of hyperparameters used to build it. Several algorithms exist for optimization of hyperparameters of ML algorithms. These include, grid search, random search, and Bayesian optimization algorithm. We optimized the performance of models using Bayesian optimization algorithm. The most important set of parameters for each ML model were chosen and optimized.

### 4.6. Performance Metrics

We used F1-score, accuracy, precision, and specificity as performance metrics. We al-so recorded the bias in the prediction of the models. Since the optimization problem was solved as a minima problem, we used (1-F1 score) as the objective function to be minimized. The positive value of bias indicates that the predicted value is higher than the true value while the negative bias shows that the predicted value is lower than true value. 

### 4.7. Python Libraries

The hyperparameters were optimized using scikit-optimize library which implemented the Bayesian optimization algorithm. The machine learning pipeline—from data preprocessing to building and training of models, prediction of gene expression values, and analysis of results—was performed using AI4Water, which is a python-based framework for performing the aforementioned tasks [13].

### 4.8. Code Availability

The code to reproduce the results presented in this article is available at GitHub repository (link: https://github.com/Asadmalic/antibiotic_prediction) (accessed on 10 November 2022).

## Figures and Tables

**Table 1:** Metrics comparison of ten different ML classification models. Models were compared on the basis of their performance to predict gene expression values of meropenem and ceftazidime on the test datasets.

Name of Model	Meropenem			Ceftazidime		
	F1-Score	Accuracy	Specificity	F1-Score	Accuracy	Specificity
CatBoostClassifier	0.4220	0.730	0.5	0.594	0.595	0.598
GaussianProcessClassifier	0.212	0.269	0.5	0.343	0.523	0.5
GradientBoostingClassifier	0.4220	0.730	0.5	0.322	0.476	0.5
HistGradientBoostingClassifier	0.589	0.707	0.576	0.570	0.571	0.5704
KNeighborsClassifier	0.629	0.709	0.629	0.618	0.619	0.618
LGBMClassifier	0.491	0.651	0.4987	0.583	0.583	0.584
RandomForestClassifier	0.422	0.730	0.5	0.602	0.607	0.603
RidgeClassifier	0.629	0.565	0.575	0.652	0.654	0.652
XGBClassifier	0.527	0.617	0.528	0.551	0.559	0.554
XGBRFClassifier	0.519	0.606	0.520	0.475	0.476	0.476

## Data Availability

The data used in this study were extracted from NCBI and can be found at: 1. https://www.ncbi.nlm.nih.gov/geo/query/acc.cgi?acc=GSE123544 (accessed on 6 April 2022). 2. https://www.ncbi.nlm.nih.gov/sra/?term=PRJNA526797 (accessed on 6 April 2022).

## Supplementary Materials

The following supporting information can be downloaded at: https://www.mdpi.com/article/10.3390/antibiotics11111593/s1, Figure S1. Bar charts showing importance of top 30 features out of 6000 features for the Ceftazidime, Meropenem and Ciprofloxacin. Figure S2. Confusion matrix for the gene expression values of P. aeruginosa isolates with a threshold set at 0.5. Rows represent the true transcriptomic values and columns represent the predictions; Figure S3. The comparison of specificity of ten classification models used for gene expression prediction for meropenem; Figure S4. The comparison of accuracy of ten classification models used for gene expression prediction for meropenem; Figure S5. The comparison of specificity of ten classification models used for gene expression prediction for ceftazidime; Figure S6. The comparison of accuracy of ten classification models used for gene expression prediction for ceftazidime; Figure S7. The comparison of accuracy of ten classification models used for gene expression prediction for Ciprofloxa-cin; Figure S8. The comparison of specificity of ten classification models used for gene expression prediction for Ciproflox-acin; Figure S9. (a). Receiver operating characteristic (ROC) curves for Catboost Classifier in case of ciprofloxacin. AUC val-ue is also shown in the figure. (b). Receiver operating characteristic (ROC) curves for GradientBoosting Classifier in case of ciprofloxacin. AUC value is also shown in the figure. (c). Receiver operating characteristic (ROC) curves for HitsGra-dientBoosting Classifier in case of ciprofloxacin. AUC value is also shown in the figure. (d) Receiver operating charac-teristic (ROC) curves for RandomForest Classifier in case of ciprofloxacin. AUC value is also shown in the figure; Figure S10. (a). Receiver operating characteristic (ROC) curves for Catboost Classifier in case of Ceftazidime. AUC value is also shown in the figure. (b). Receiver operating characteristic (ROC) curves for GradientBoosting Classifier in case of Ceftazidime. AUC value is also shown in the figure. (c). Receiver operating characteristic (ROC) curves for HitsGradi-entBoosting Classifier in case of Ceftazidime. AUC value is also shown in the figure. (d). Receiver operating characteris-tic (ROC) curves for Randomforest Classifier in case of Ceftazidime. AUC value is also shown in the figure; Figure S11 (a). Receiver operating characteristic (ROC) curves for Catboost Classifier in case of meropenem. AUC value is also shown in the figure. (b). Receiver operating characteristic (ROC) curves for Gradientboosting Classifier in case of meropenem. AUC value is also shown in the figure. (c). Receiver operating characteristic (ROC) curves for HitsGradientboosting Classifier in case of meropenem. AUC value is also shown in the figure. (d) Receiver operating characteristic (ROC) curves for Randomforest Classifier in case of meropenem. AUC value is also shown in the figure; Table S1. Metrics comparison of ten different ML classification models. Models were compared on the basis of their performance to predict gene expression values of ciprofloxacin on the test datasets.
