# Supplementary material for: Application of Decision-Tree-Based Machine Learning Algorithms for Prediction of Antimicrobial Resistance
Source: Antibiotics (Basel). 2022 Nov 10;11(11):1593. doi: 10.3390/antibiotics11111593 (PMC9686960; doi:10.3390/antibiotics11111593)
Supplement: Supplementary file 1 [file antibiotics-11-01593-s001.zip › antibiotics-1928820-supplementary.pdf]

antibiotics

Application of Decision Tree Based Machine Learning Algorithms for Prediction of Antimicrobial Resistance

Muhammad Yasir[1, 2,†,*], Asad Mustafa Karim[3, †,*], Sumera Kausar Malik[4], Amal A. Bajaffer[1], Esam I. Azhar[1,]

[1] Special Infectious Agents Unit, King Fahd Medical Research Center, King Abdulaziz University, Jeddah 21589, Saudi Arabia
[2] Department of Medical Laboratory Sciences, Faculty of Applied Medical Sciences, King Abdulaziz University, Jeddah 21589, Saudi Arabia
[3] Graduate School of Biotechnology, College of Life Sciences, Kyung Hee University, Yongin 17104, South Korea;
[4] Department of Bioscience and Biotechnology, The University of Suwon, Hwaseong City, Gyeonggi-do 18323, South Korea
* Correspondence: yasirkhattak.mrl@gmail.com (M.Y.); asadmustafa8@gmail.com (A.M.K.)

Figure S1. Bar charts showing importance of top 30 features out of 6,000 features for the Ceftazidime, Meropenem and Ciprofloxacin.

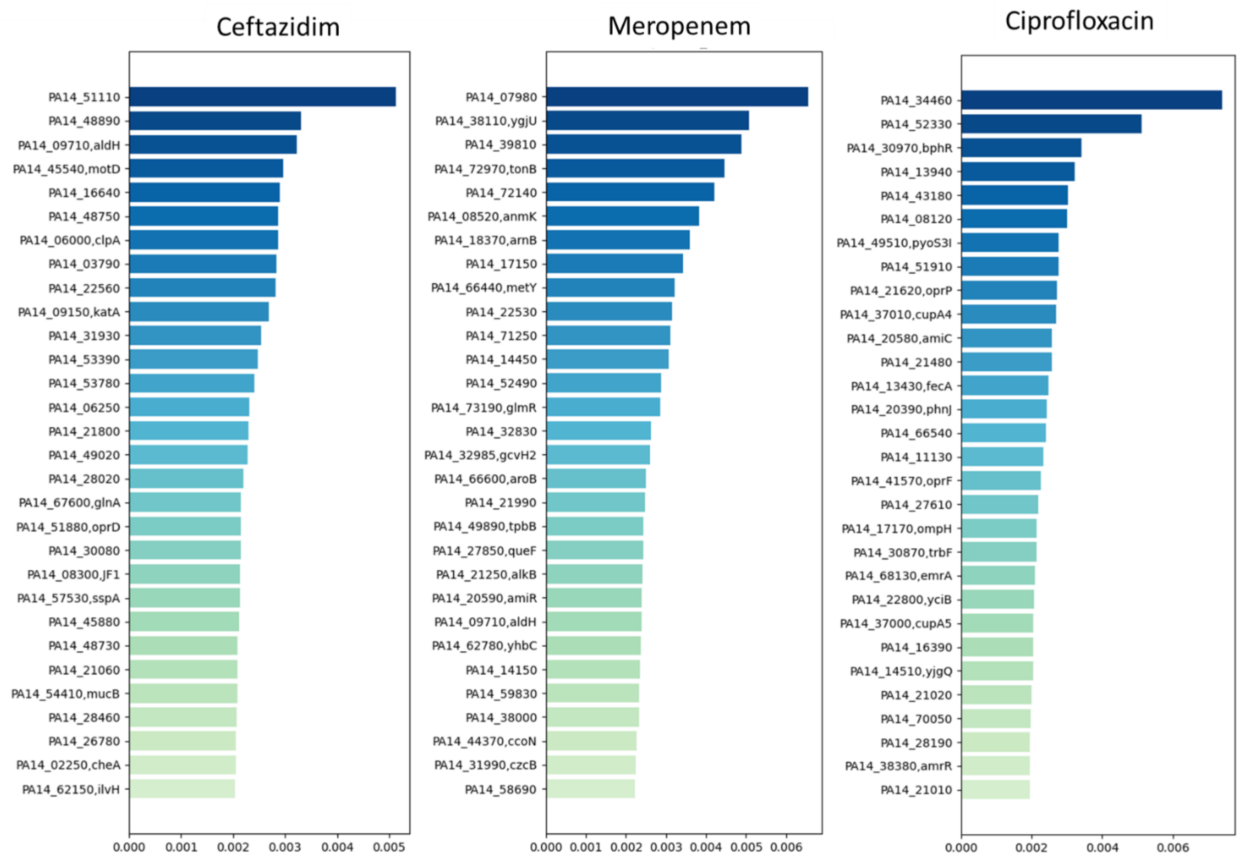

Figure S2. Confusion matrix for the gene expression values of *P. aeruginosa* isolates with a threshold set at 0.5. Rows represent the true transcriptomic values and columns represent the predictions.

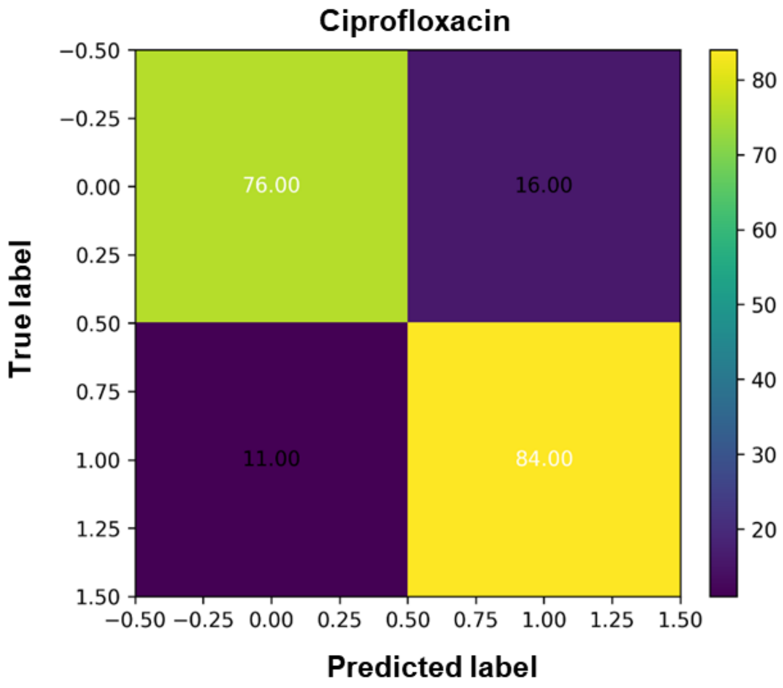

Figure S3. The comparison of specificity of ten classification models used for gene expression prediction for meropenem.

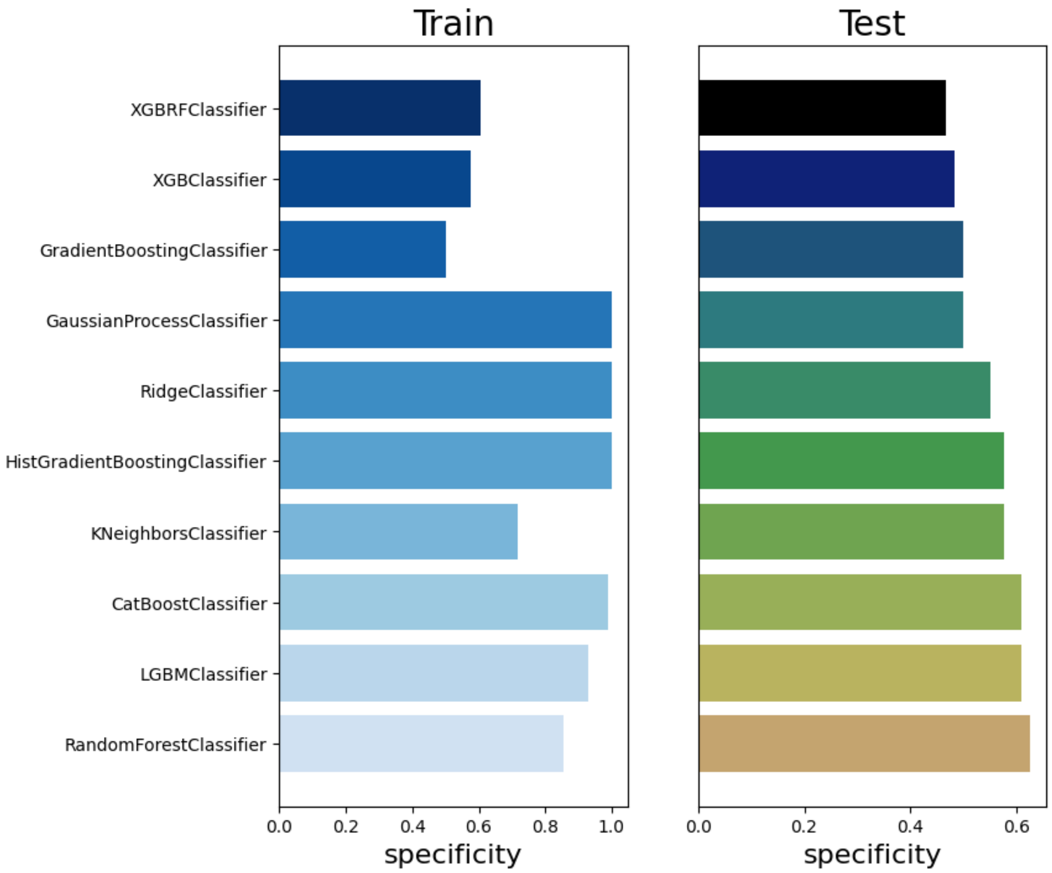

Figure S4. The comparison of accuracy of ten classification models used for gene expression prediction for meropenem.

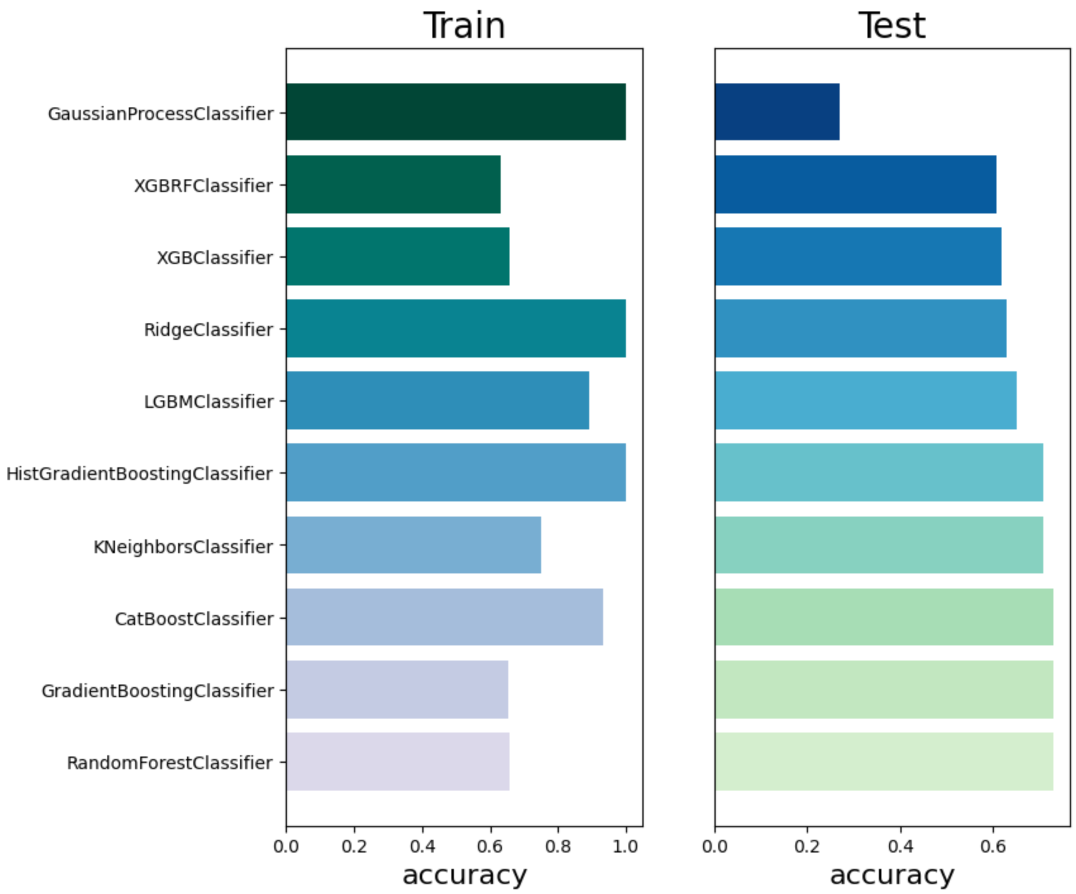

Figure S5. The comparison of specificity of ten classification models used for gene expression prediction for ceftazidime.

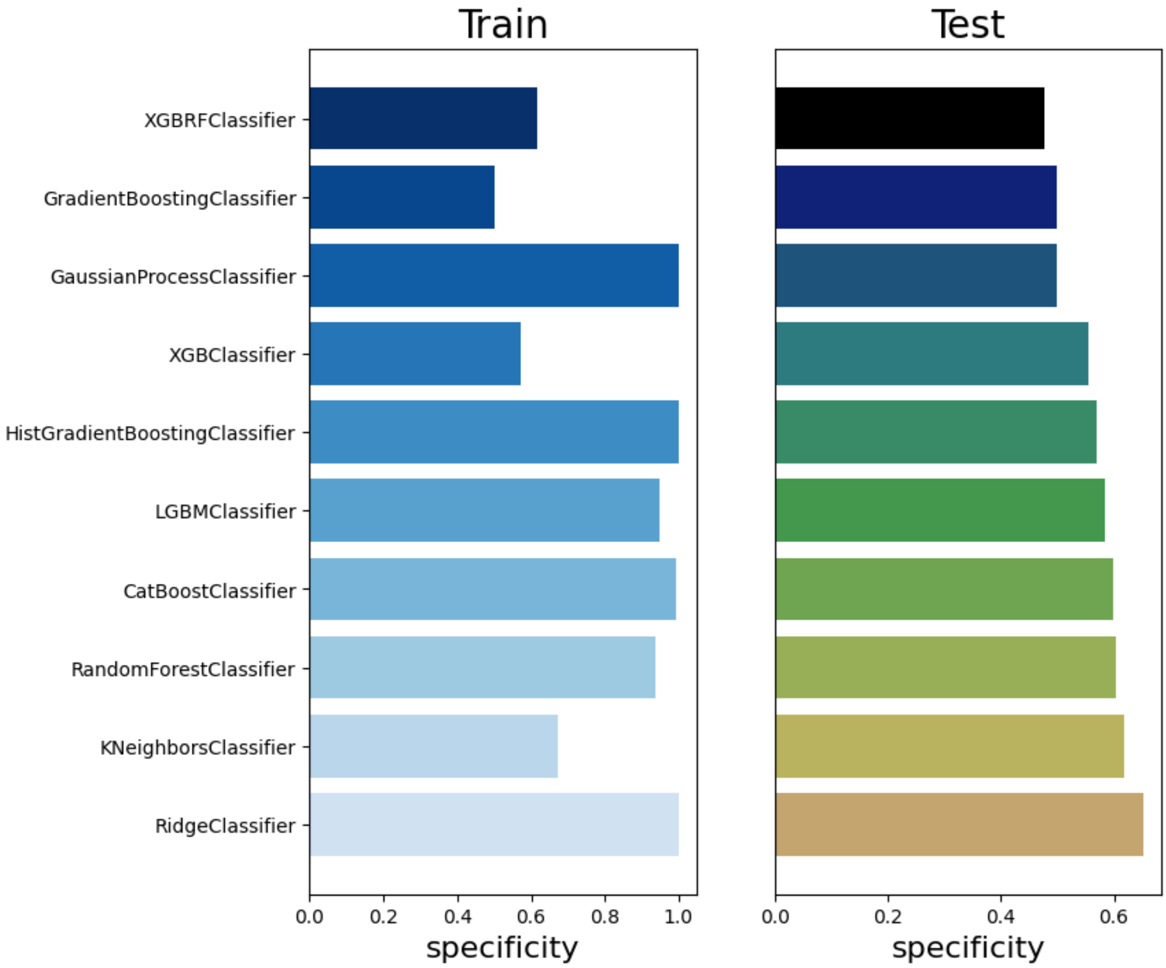

Figure S6. The comparison of accuracy of ten classification models used for gene expression prediction for ceftazidime.

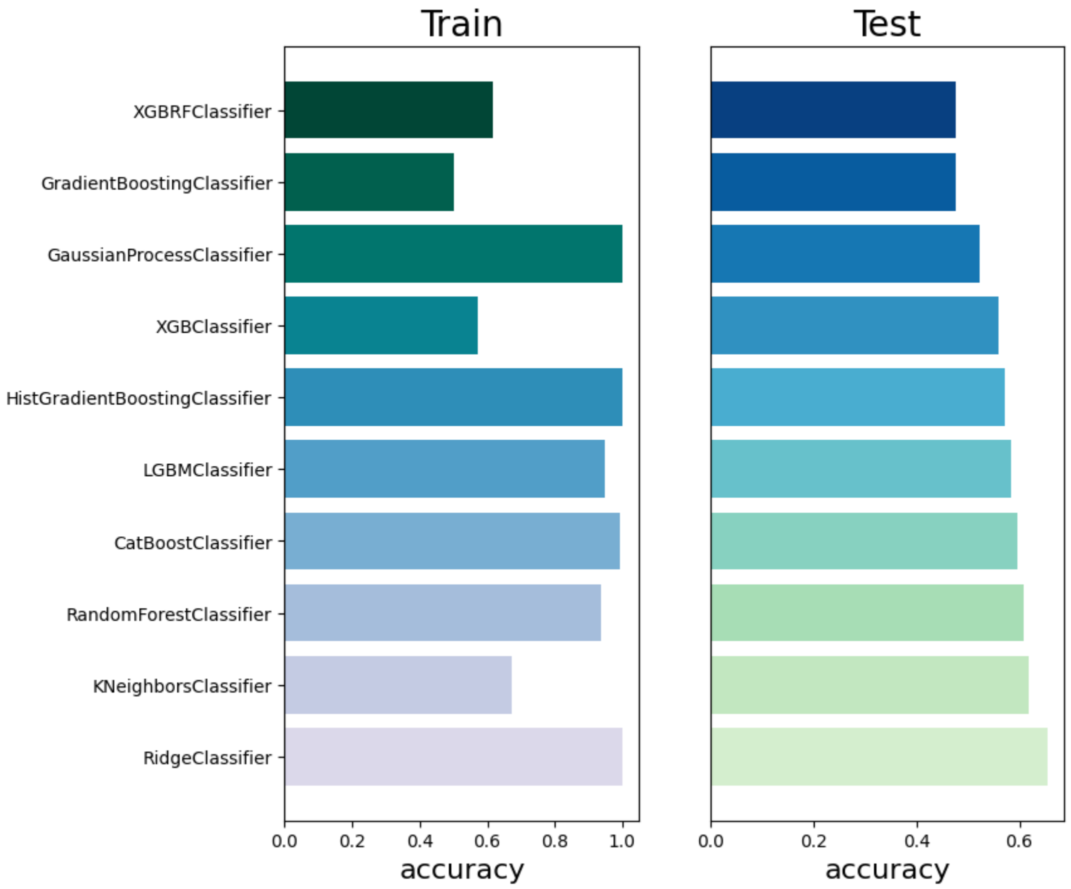

Figure S7. The comparison of accuracy of ten classification models used for gene expression prediction for Ciprofloxacin.

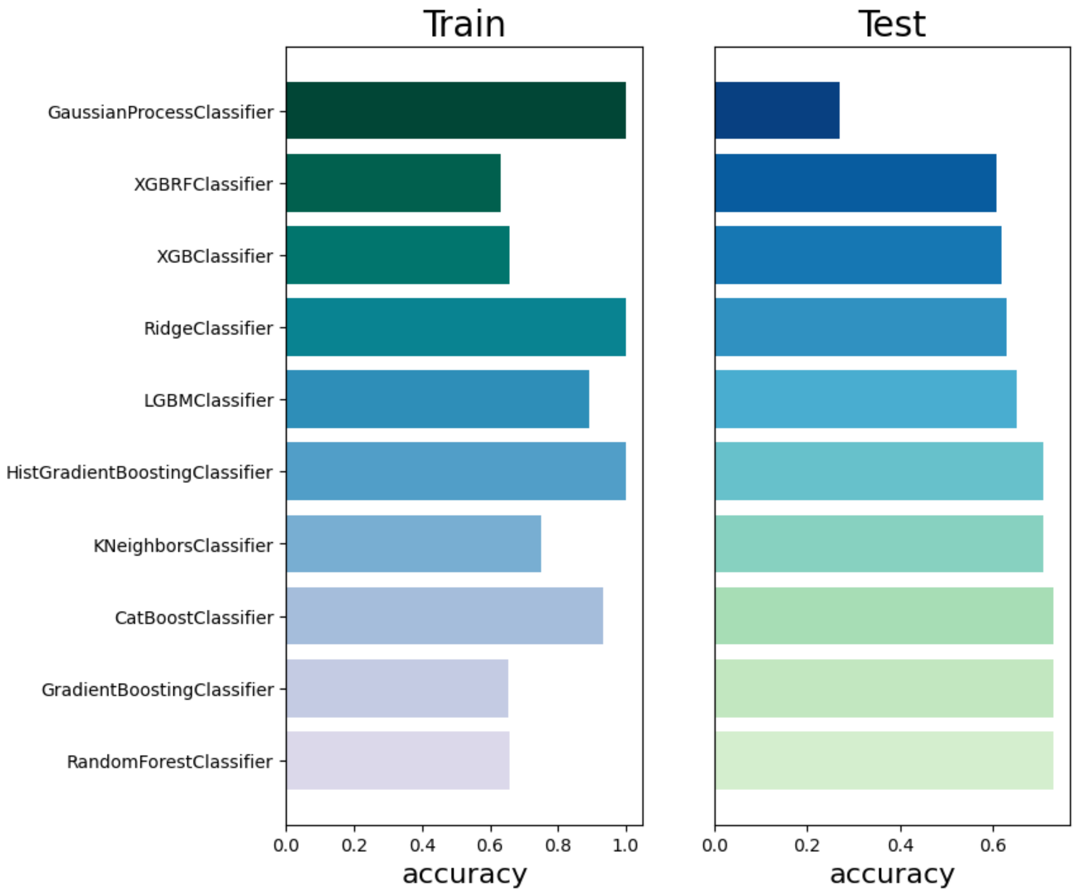

Figure S8. The comparison of specificity of ten classification models used for gene expression prediction for Ciprofloxacin.

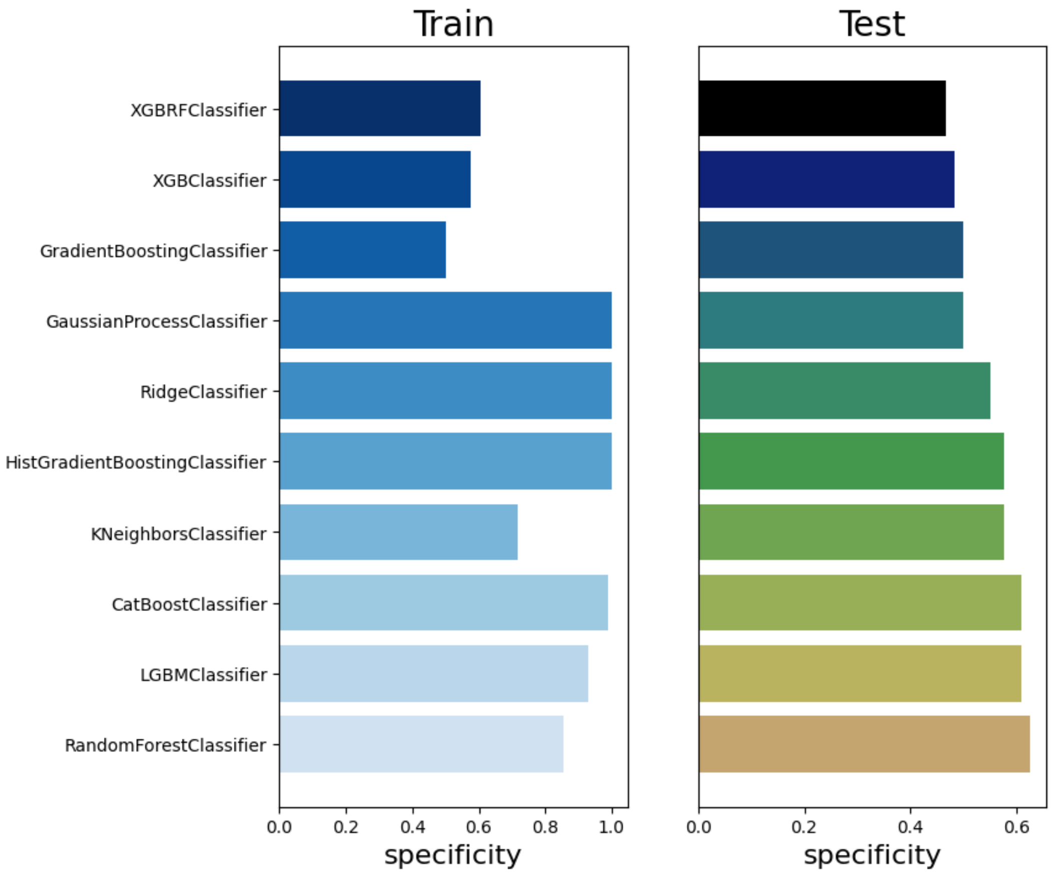

Figure S9. (a). Receiver operating characteristic (ROC) curves for Catboost Classifier in case of ciprofloxacin. AUC value is also shown in the figure. **(b).** Receiver operating characteristic (ROC) curves for GradientBoosting Classifier in case of ciprofloxacin. AUC value is also shown in the figure. **(c)**. Receiver operating characteristic (ROC) curves for HitsGradientBoosting Classifier in case of ciprofloxacin. AUC value is also shown in the figure. **(d)** Receiver operating characteristic (ROC) curves for RandomForest Classifier in case of ciprofloxacin. AUC value is also shown in the figure.

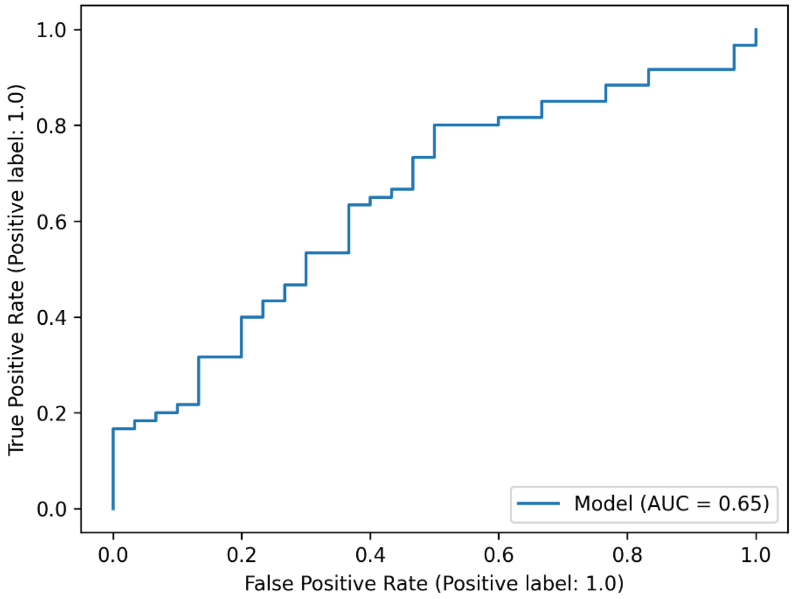

(**a**)

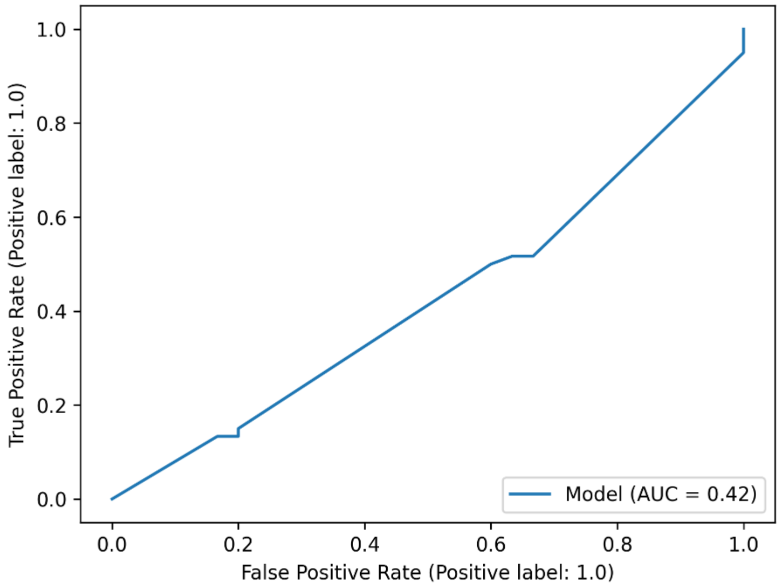

(**b**)

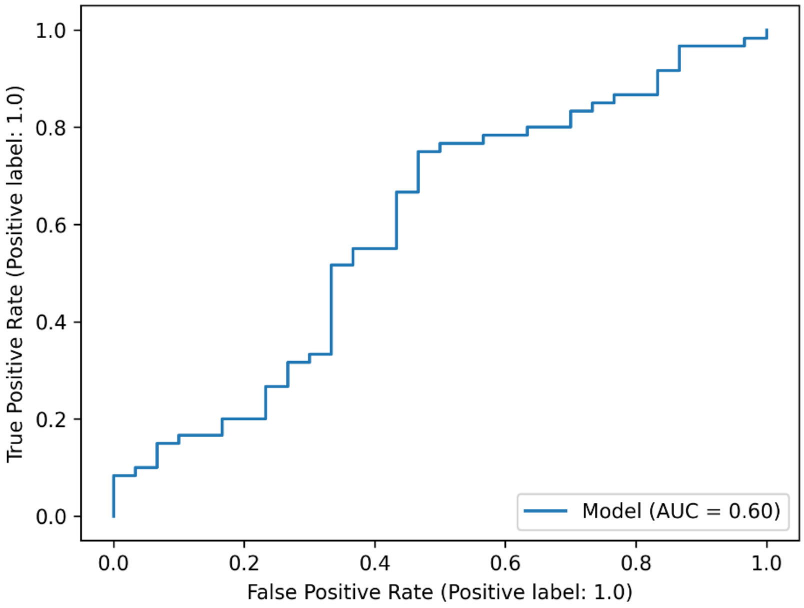

1.0
0.8
0.6
0.4
0.2
0.0
True Positive Rate (Positive label: 1.0)
Model (AUC = 0.60)
0.0
0.2
0.4
0.6
0.8
1.0
False Positive Rate (Positive label: 1.0)

(**c**)

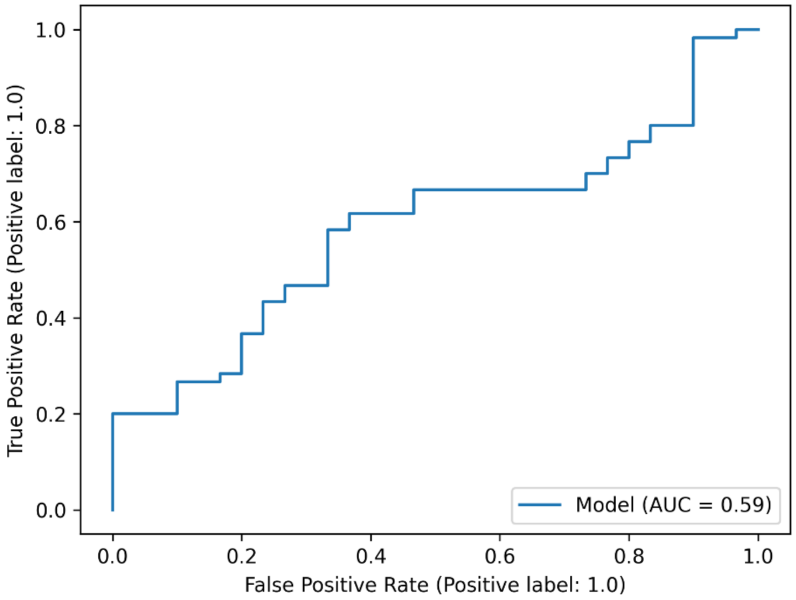

1.0
0.8
0.6
0.4
0.2
0.0
True Positive Rate (Positive label: 1.0)
Model (AUC = 0.59)
0.0
0.2
0.4
0.6
0.8
1.0
False Positive Rate (Positive label: 1.0)

(**d**)

Figure S10. (a). Receiver operating characteristic (ROC) curves for Catboost Classifier in case of Ceftazidime. AUC value is also shown in the figure. **(b)**. Receiver operating characteristic (ROC) curves for GradientBoosting Classifier in case of Ceftazidime. AUC value is also shown in the figure. **(c)**. Receiver operating characteristic (ROC) curves for HitsGradientBoosting Classifier in case of Ceftazidime. AUC value is also shown in the figure. **(d)**. Receiver operating characteristic (ROC) curves for Randomforest Classifier in case of Ceftazidime. AUC value is also shown in the figure.

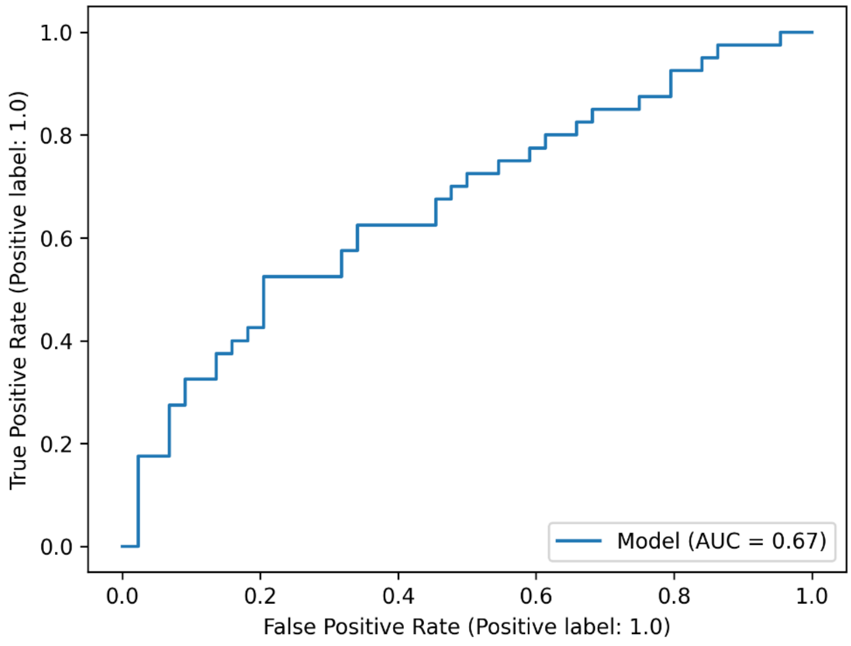

(**a**)

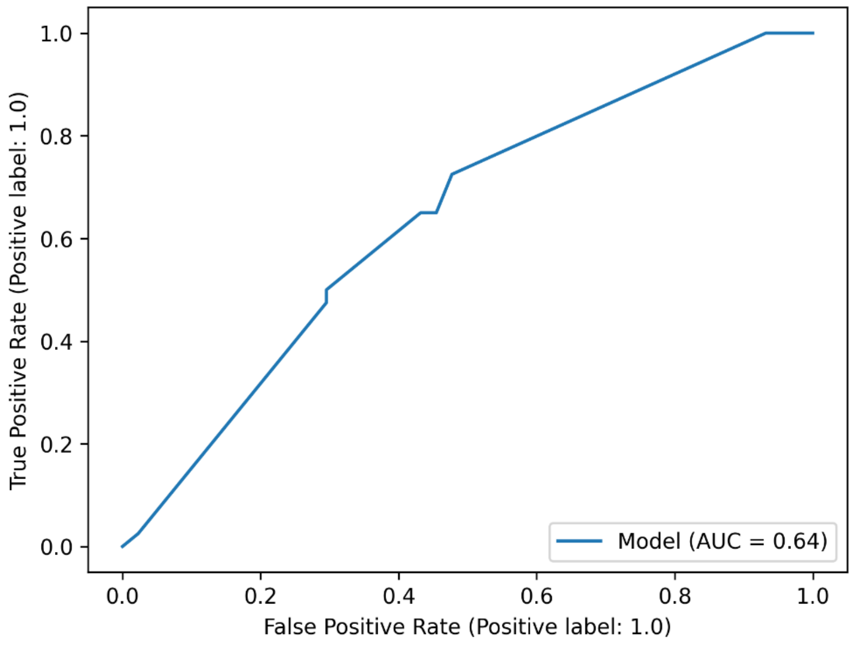

(**b**)

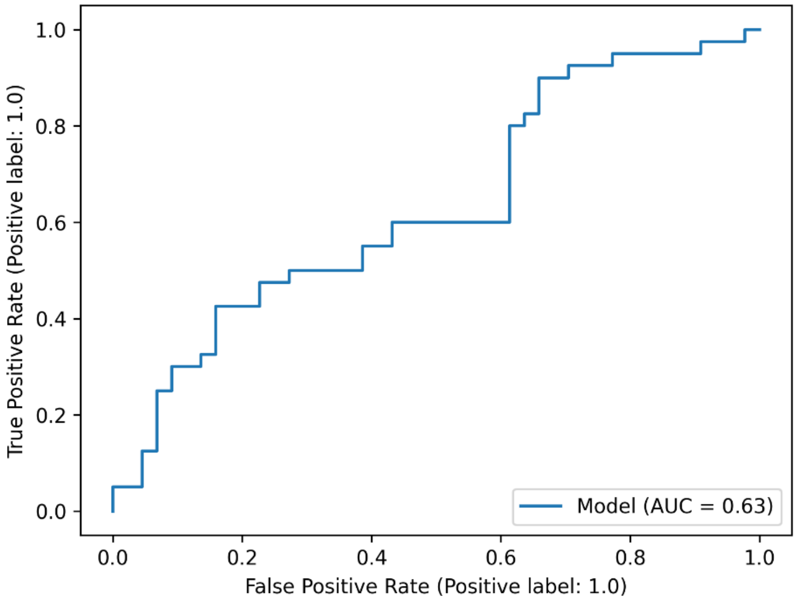
1.0
0.8
0.6
0.4
0.2
0.0
True Positive Rate (Positive label: 1.0)
Model (AUC = 0.63)
0.0
0.2
0.4
0.6
0.8
1.0
False Positive Rate (Positive label: 1.0)

(**c**)

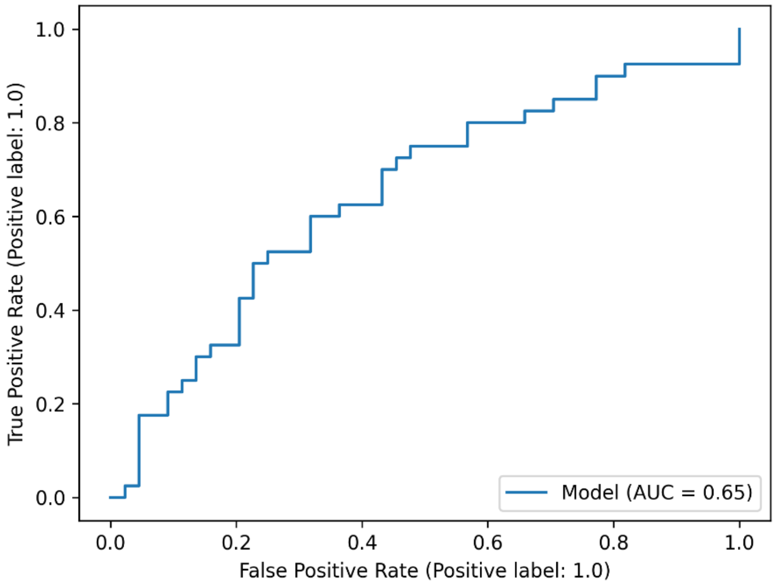
1.0
0.8
0.6
0.4
0.2
0.0
True Positive Rate (Positive label: 1.0)
Model (AUC = 0.65)
0.0
0.2
0.4
0.6
0.8
1.0
False Positive Rate (Positive label: 1.0)

(**d**)

Figure S11 (a). Receiver operating characteristic (ROC) curves for Catboost Classifier in case of meropenem. AUC value is also shown in the figure. **(b)**. Receiver operating characteristic (ROC) curves for Gradientboosting Classifier in case of meropenem. AUC value is also shown in the figure. **(c)**. Receiver operating characteristic (ROC) curves for HitsGradientboosting Classifier in case of meropenem. AUC value is also shown in the figure. **(d)** Receiver operating characteristic (ROC) curves for Randomforest Classifier in case of meropenem. AUC value is also shown in the figure.

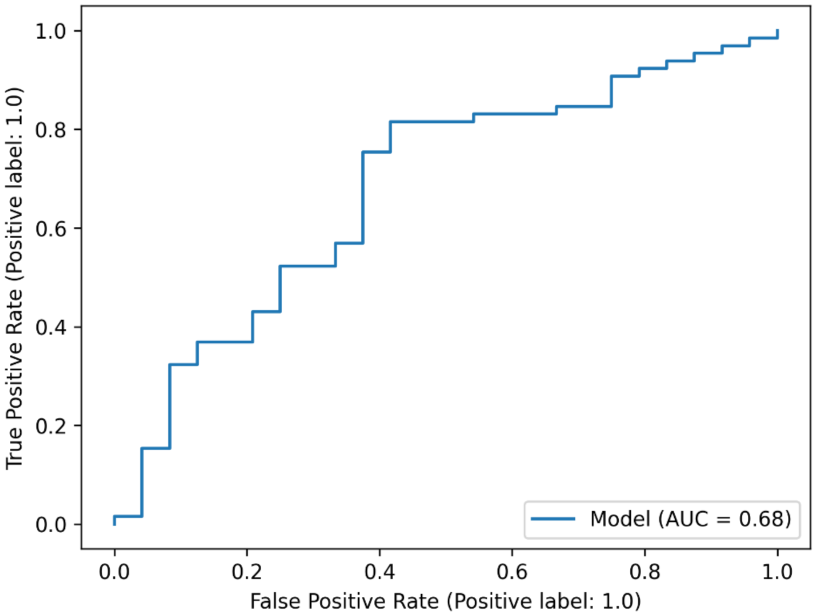

(**a**)

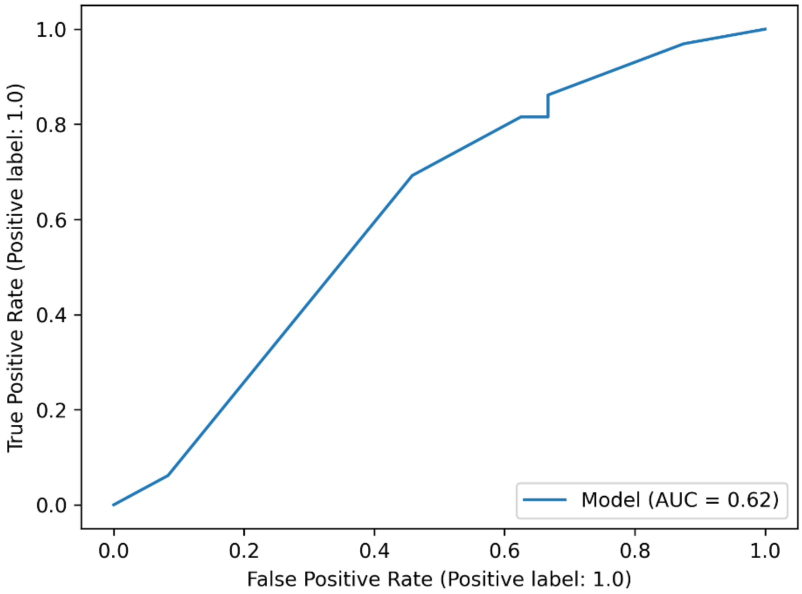

(**b**)

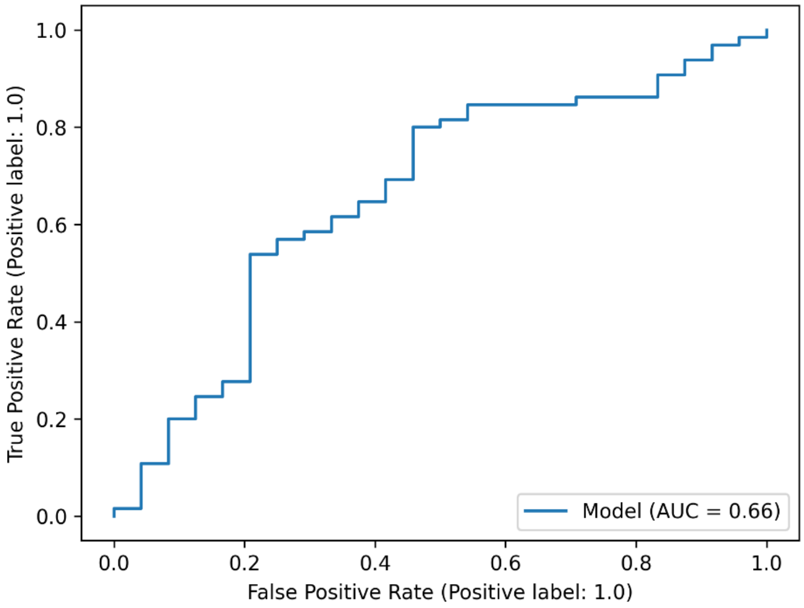
True Positive Rate (Positive label: 1.0)
False Positive Rate (Positive label: 1.0)
Model (AUC = 0.66)
0.0
0.2
0.4
0.6
0.8
1.0

(**c**)

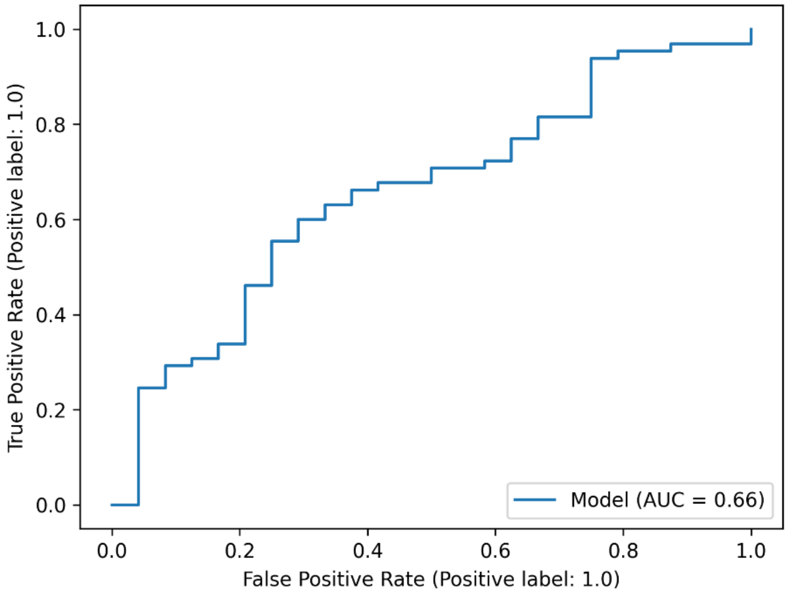
True Positive Rate (Positive label: 1.0)
False Positive Rate (Positive label: 1.0)
Model (AUC = 0.66)
0.0
0.2
0.4
0.6
0.8
1.0

(**d**)

Table S1. Metrics comparison of ten different ML classification models. Models were compared on the basis of their performance to predict gene expression values of ciprofloxacin on the test datasets.

	F1-score	precision	specificity
CatBoostClassifier	0.586	0.596	0.608
GaussianProcessClassifier	0.25	0.16666	0.5
GradientBoostingClassifier	0.4	0.3333	0.5
HistGradientBoostingClassifier	0.554	0.566	0.575
KNeighborsClassifier	0.539	0.567	0.575
LGBMClassifier	0.598	0.598	0.608
RandomForestClassifier	0.6	0.611	0.625
RidgeClassifier	0.537	0.5446	0.55
XGBClassifier	0.395	0.4799	0.4833
XGBRFClassifier	0.4650	0.4694	0.4666
